# Delay minimization based uplink resource allocation for device-to-device communications considering mmWave propagation

**DOI:** 10.7717/peerj-cs.462

**Published:** 2021-04-08

**Authors:** Marcus V.G. Ferreira, Flávio Henrique Teles Vieira

**Affiliations:** 1 Universidade Federal de Goiás, Instituto de Informática, Goiânia, Goiás, Brazil; 2 Escola de Engenharia Elétrica, Mecânica e de Computação, Universidade Federal de Goiás, Goiânia, Goiás, Brazil

**Keywords:** 5G, D2D, mmWaves, Delay, Multi-sharing

## Abstract

This paper addresses the resource allocation problem in multi-sharing uplink for device-to-device (D2D) communication, one aspect of 5G communication networks. The main advantage and motivation in relation to the use of D2D communication is the significant improvement in the spectral efficiency of the system when exploiting the proximity of communication pairs and reusing idle resources of the network, mainly in the uplink mode, where there are more idle available resources. An approach is proposed for allocating resources to D2D and cellular user equipments (CUE) users in the uplink of a 5G based network which considers the estimation of delay bound value. The proposed algorithm considers minimization of total delay for users in the uplink and solves the problem by forming conflict graph and by finding the maximal weight independent set. For the user delay estimation, an approach is proposed that considers the multifractal traffic envelope process and service curve for the uplink. The performance of the algorithm is evaluated through computer simulations in comparison with those of other algorithms in the literature in terms of throughput, delay, fairness and computational complexity in a scenario with channel modeling that describes the propagation of millimeter waves at frequencies above 6 GHz. Simulation results show that the proposed allocation algorithm outperforms other algorithms in the literature, being highly efficient to 5G systems.

## Introduction

Wireless communication applications have become very popular, and several technologies were developed to improve the quality of service (QoS) during last decades. The increasing demand for wireless traffic through various applications such as augmented reality, machine-to-machine (M2M) communication, internet of things (IoT), among others, has driven telecommunications operators to increase the bandwidth of their systems and provide service with lower latency and higher throughput. However, there are several limitations of the system that hinder the increase in bandwidth, making it necessary to think about more advanced cellular communication systems with better spectral efficiency to support the growing number of devices in the network (Mishra, Pandey & Biswash, 2016).

Among the strands of 5G communication networks, one that stands out is device-to-device (D2D) communication. The D2D communication was introduced in LTE-A (long term evolution advanced). However, this technology was in the background for a long time, not being used much by telecommunications operators. In this scenario, the D2D devices communicate with each other with low interference from the base station (BS), depriving the BS of traffic overload (Mishra, Pandey & Biswash, 2016). Most works related to D2D communication deal with sharing resources in the uplink instead of the downlink, due to the interest in taking advantage of the asymmetric characteristic of data traffic in Internet networks. More specifically, Internet traffic is much higher on the downlink, thus it seems reasonable to suppose that there is a greater availability of idle resources to be explored in the uplink than in the downlink (Pan et al., 2018).

For resource sharing on the uplink (Sun et al., 2013) has developed an heuristic that guarantees a maximum number of device pairs communicating with each other with minimal interference, denoted as greedy resource allocation (GRA) algorithm. The authors show that the problem is essentially an allocation problem and proposed an optimal algorithm based on the Hungarian method. However, the algorithm proposed by Sun et al. (2013) was not developed for 5G networks, where can exist a large number of devices in the network. Moreover, the authors considered a single-sharing scenario where each allocated resource block (RB) can be reused by only one pair of devices. In 5G networks, it is desirable that any RB allocated to a device can be reused by several devices, which characterizes multi-sharing communication.

Ciou et al. (2015) then proposed a method based on the algorithm described by Sun et al. (2013) to solve the multi-sharing resources allocation problem in order to increase the throughput of the system and to guarantee the signal-to-interference-plus-noise ratio (SINR) requirements, denoted as greedy throughput maximization plus (GTM+). The authors formulated the multi-sharing allocation problem and proved that it is a non-deterministic polynomial-time hard problem. More specifically, Ciou et al. (2015) proposed an algorithm with a fast and efficient solution that explores conflict graphs and maximal weight independent, showing that it outperforms other existing schemes.

This paper proposes a resource allocation algorithm for multi-sharing communication which considers the estimation of delay information of the uplink in order to optimize overall system performance considering system delay minimization. In this context of multi-sharing communication, the delay is considered as an essential QoS parameter, especially for real-time applications with variable transmission rates and specified bandwidth requirements, such as videoconferencing services. To this end, first an approach is proposed to estimate delay bound using envelope process for traffic flows and a service curve for the uplink transmission. The proposed algorithm uses concepts such as conflict graph and maximal weight independent set. However, different from the algorithms of the previous mentioned works, we propose an algorithm to solve the multi-sharing resource allocation problem that considers the minimization of the estimated delay information of the uplink.

The implemented scenario simulation considers the propagation of millimeter waves (mmWaves) above 6 GHz, an important characteristic of 5G networks which leads to improvements in throughput and latency for network users. Simulation results show that the proposed algorithm provides considerable gains in terms of throughput, delay and fairness to the performance of the considered 5G scenario, presenting lower computational complexity compared to some optimization heuristics.

The remainder of the paper is organized as follows: “System Model and Problem Formulation” describes the system model and formulates the multi-sharing resource allocation problem. “Related Works” presents related works. “Delay Minimization Based D2D Resource Allocation Algorithm” presents the algorithm proposed in this paper. “Simulations and Results” presents the performance evaluation of the algorithms. “Conclusion” presents the final considerations.

### System model and problem formulation

**Figure 1:**
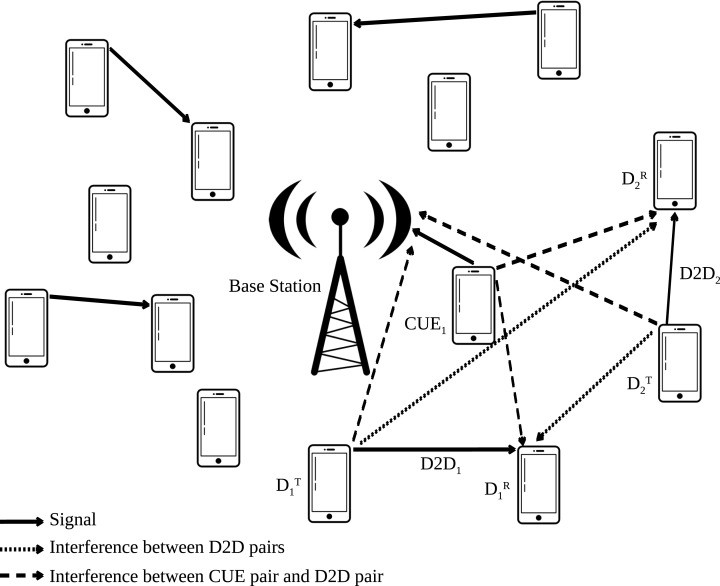
System model of D2D communications reusing the uplink resources of CUES.

Figure 1 represents the system model of D2D communications which reuse the uplink resources of equipments in wireless networks. In these systems, there are two types of equipments: pairs of D2D user equipments (DUEs) and cellular user equipments (CUEs). The first one reuse radio resource allocated to the CUEs and communicate directly without communication load on the base station (BS).

This system is composed of *M* CUEs, including real and virtual CUEs, *N* DUEs, *K* idle resource blocks (RBs) scheduled for transmission during a transmission time interval (TTI) and *M* − *K* real CUEs. The RBs correspond to the minimum allocation unit in a wireless communication system, and each idle RB, not used by any real CUEs, is regarded as a virtual CUE with zero transmit power and no signal-to-interference-plus-noise ratio (SINR) requirement. The CUEs are denoted by *C*_1_,*C*_2_,…,*C*_*M*_ and the DUEs by *D*_1_,*D*_2_,…,*D*_*N*_. The DUE pair tdransmitter is denoted by *D*_*n*,*Tx*_ and the receiver by *D*_*n*,*Rx*_. In order to facilitate the comprehension of the proposed approach and of the considered system model, Table 1 presents the variables and parameters considered in this paper.

**Table 1:** Notation table.

*M*	Number of CUEs
*N*	Number of DUEs
*K*	Number of idle resource blocks
*C*_1_,*C*_2_,…,*C*_*M*_	CUEs denotation
*D*_1_,*D*_2_,…,*D*_*N*_	DUEs denotation
*W*_*m*_	Allocation bandwidth for CUE *m*
Θ_*n*_	Set of CUEs that share their RBs with DUE pair *n*
Δ_*m*_	Set of DUE pairs that reuse the RBs allocated to CUE *m*
*P*_*m*_	Transmit power for CUE *m*
*G*_*mB*_	Channel gain between CUE *m* and the BS
σ^2^_*m*_	Noise power for CUE *m*
*P*_*n*_	Transmit power for DUE pair *n*
*G*_*nB*_	Channel gain between DUE pair *n* transmitter and the BS
*γ*_*m*_	SINR threshold required by CUE *m*
*G*_*nn*_	Channel gain between the DUE pair *n* two ends
σ^2^_*n*_	Noise power for DUE pair *n*
*G*_*mn*_	Channel gain from CUE *m* to DUE pair *n*
*G*_*n*′*n*_	Channel gain from *D*_*n*′,*Tx*_ to *D*_*n*,*Rx*_
*F*	Total system throughput
Â (*t*)	Maximum value of a flow *A*(*t*) in the time slot [*s*, *s* + *t*]
Â_MFBAP_(*t*)	Multifractal bounded arrival process
*H*(*t*)	Hölder exponent
*t*	Instant of time
ā	Mean of the incoming traffic
σ	Standard deviation of the incoming traffic
*k*	Constant related to the probability of violation of the envelope process
*B*	Buffer size
$\hat{d}$	Estimated delay
*S*	Generalized service curve of an OFDM system
*c*	Average service rate on the system server
*N*_*S*_	Number of time slots *T* per complete cycle *P* given by $P=\left[\frac{t}{N_{S}T}\right]$
Γ_*m*_	Set of unmarked pair *m*
Δ_*m*′_	Set of DUE pairs that will reuse RBs of CUE *m*′
*G*_*m*′_	Conflict graph formed by DUE pairs representing the vertices
*U*	Set of unmarked groups
*I*_*m*′_	Maximum tolerable interference for CUE *m*
*C*_*m*′_	Set of DUEs in Δ_*m*′_ sorted by their interference

Before sharing the RBs to DUEs during transmission, it is pre-allocated a set of uplink RBs for each CUE. The allocated bandwidth denoted by *W*_*m*_ for each CUE *m* is proportional to the number of allocated RBs. Each CUE can share its RBs with several pairs of DUEs, making it possible a large number of simultaneous connections between DUEs. This characteristic describes the multi-sharing resource allocation.

The CUE and DUE relationship is specified thought a set denoted as Θ_*n*_, which represents the set of CUEs that share their RBs with DUE pair *n*, and a set denoted as Δ_*m*_, which represents the set of the DUE pairs that reuse the RBs allocated to CUE *m*. These sets Δ_1_,Δ_2_,…,Δ_*M*_ are subsets of {1,2,...,N}.

The set of DUEs only can reuse uplink RBs from CUE *m* if the interference on *m*’s transmission obey its SINR requirement. The received SINR for any CUE m∈{1,2,...,M} must be superior to the following threshold:

(1) $$\frac{P_{m}G_{mB}}{\sigma_{m}^{2}+\sum_{n\in \Delta_{m}}P_{n}G_{nB}}\geq \gamma_{m},$$

where *P*_*m*_ is the transmit power for CUE *m*, *G*_*mB*_ is the channel gain between CUE *m* and the BS, σ^2^_*m*_ is the noise power for CUE *m*, *P*_*n*_ is the transmit power for DUE pair *n*, *G*_*nB*_ is the channel gain between DUE pair *n* transmitter and the BS and *γ*_*m*_ is the SINR threshold required by CUE *m*.

There are also SINR requirements for the DUE pairs. Certain RBs can be reused by a DUE pair *n* only if the received SINR is higher than:

(2) $$\frac{P_{n}G_{nn}}{\sigma_{n}^{2}+P_{m}G_{mn}+\sum_{n^{\prime }\in \Delta_{m}-\left\{n\right\}}P_{n^{\prime }}G_{n^{\prime }n}}\geq \gamma_{n},\forall m\in \Theta_{n},$$

where *P*_*n*_ is the transmit power for DUE pair *n*, *G*_*nn*_ is the channel gain between the DUE pair *n* two ends, σ^2^_*n*_ is the noise power for DUE pair *n*, *P*_*m*_ is the transmit power for CUE *m*, *G*_*mn*_ is the channel gain from CUE *m* to DUE pair *n*, *G*_*n*′*n*_ is the channel gain from *D*_*n*′,*Tx*_ to *D*_*n*,*Rx*_ and *γ*_*n*_ is the SINR threshold for DUE pair *n*.

It is assumed in this work that the BS knows the channel properties such as transmit power, channel gain and noise power of the communication link for CUEs *m* and DUE pairs *n*, i.e., the BS knows the channel state information (CSI) and the SINR threshold for each user.

The multi-sharing resource allocation problem objective is determine which DUEs reuse RBs from CUEs such that the total system throughput *F* is maximized. The total system throughput *F* is defined as the sum of all CUEs’ and DUEs’ Shannon capacities and is formulated as follows:

(3) $$\begin{array}{l} F=\underset{\Delta_{1},\Delta_{2},\ldots ,\Delta_{M}}{\max}\sum_{m=1}^{M}\{W_{m}\log2(1+\frac{P_{m}G_{mB}}{\sigma_{m}^{2}+\sum_{n\in \Delta_{m}}P_{n}G_{nB}})+\\ \sum_{n\in \Delta_{m}}W_{m}\log2(1+\frac{P_{n}G_{nn}}{\sigma_{n}^{2}+P_{m}G_{mn}+\sum_{n^{\prime }\in \Delta_{m}-\left\{n\right\}}P_{n^{\prime }}G_{n^{\prime }n}})\}, \end{array}$$

subject to:

(4) $$\sum_{n\in \Delta_{m}}P_{n}G_{nB}\leq \frac{P_{m}G_{mB}}{\gamma_{m}}-\sigma_{m}^{2},\;\forall m\in \left\{1,2,...,M-K\right\},$$

(5) $$P_{m}G_{mn}+\sum_{n^{\prime }\in \Delta_{m}-\left\{n\right\}}P_{n^{\prime }}G_{n^{\prime }n}\leq \frac{P_{n}G_{nn}}{\gamma_{n}}-\sigma_{n}^{2},\;\forall n\in \left\{1,2,\ldots ,N\right\},\;\forall m\in \Theta_{n},$$

(6) $$n\in \Delta_{m},\forall n\in \left\{1,2,\ldots ,N\right\},\forall m\in \Theta_{n},$$

where constraint (4) represents the maximum tolerable interference on *m*’s transmission and constraint (5) represent the maximum tolerable interference on *n*’s transmission.

The multi-sharing resource allocation problem described by Eq. (3) under constraints (4), (5) and (6) is non-deterministic polynomial-time hard. That is, it is NP-hard as proved in Sun et al. (2013) and Ciou et al. (2015), which justifies the need to find an efficient and fast algorithm to solve the problem, such as the one presented in this paper.

### Related works

In this section, we briefly comment about some works related to D2D communications and multi-sharing resource allocation. The greedy throughput maximization plus algorithm (GTM+) is an iterative algorithm proposed by Ciou et al. (2015) which attempt to find a solution to the multi-sharing resources allocation problem. This algorithm is based on the maximization of a utility function given in terms of the system throughput considering that RBs are reused (Ciou et al., 2015). The authors in Ciou et al. (2015) use the heuristic algorithm proposed by Basagni (2001) to obtain a maximal weight independent set, with time complexity *O*(*n*^3^). The worst-case complexity of GTM+ is *O*(*n*^4^), because in each iteration, at least one DUE pair is granted to reuse the RBs. Contrary to our proposal, the GTM+ algorithm does not consider the system delay as an optimization metric.

Zhang, Hu & Qian (2016) proposed to use a distance based power control scheme for D2D communication in order to achieve expected performance gain without causing performance degradation to the primary cellular users due to system interference. The authors applies the Poisson point process (PPP) model, a stochastic geometry model, to get tractable analysis results. Numeric results presented in simulations show that the proposed scheme is benefit for both CUEs and DUEs. The initial results demonstrate the advantages of using the power control scheme, although comparisons are not made with state-of-the-art schemes in terms of throughput and delay for resource allocation in D2D communication.

Mishra, Pandey & Biswash (2016) proposed a resource allocation scheme for D2D communication in the uplink which consist of two phases. In the first phase, if multihop communication (two-hops) is required, a relay is selected from the available relays between cell edge device and BS. The relay selection scheme selects the parameters such as battery power and reliability to minimize packet loss. In the second phase, an efficient resource allocation scheme is proposed that reduces the upload time and optimizes the number of resource blocks. In this work, we also address resource allocation scheme for D2D communication in the uplink but the throughput maximization occurs via a delay minimization based algorithm.

In Pan et al. (2018), the authors studied power control and the problem of resource allocation in D2D communication underlaying a non-orthogonal multiple access (NOMA) cellular network. The objective of the work is to maximize the throughput sum of all D2D pairs by meeting the minimum rate constraints of users and using techniques of successive interference cancelation. The optimal conditions for power control of cellular users in each subchannel are derived first. Then, it is proposed a dual-based iterative algorithm to solve the resource allocation problem. The results show that the proposed scheme outperforms the conventional scheme for the network with high data requirements. Different from Pan et al. (2018), we consider in this work an uplink of CP-OFDM (cyclic prefix - orthogonal frequency-division multiple access) based communication system. However, we also aim to enhance network QoS parameters such as throughput and delay through the application of our resource allocation approach.

Khuntia & Hazra (2019) proposed a Deep Q-learning with an extended Kalman filter (EKF) scheme to solve the channel and power allocation issue for D2D communication devices when the prior traffic information is not known to the BS. They explore an optimal policy for resource and power allocation with the aim of maximizing the total throughput of the system. The resource allocation scheme comprises of four phases, i.e., cell splitting, clustering, queuing model and channel allocation simultaneously with power allocation. It is used EKF together with Deep Q-Network to incorporate weight uncertainty of the Q-function as well as the state uncertainty during transition, helping the learner in achieving an optimal policy. The authors show the advantage of the resource sharing scheme over other existing schemes through numerical simulations.

The author proposed in Sreedevi & Rama Rao (2019) to use reinforcement-learning based latency controlled D2D connectivity (RL-LCDC) algorithm and its Q-Learning approach in an indoor D2D communication network for 5G connectivity with minimum latency. The algorithm discovers the neighbors, decides the D2D link and adaptively controls the communication range for maximum network connectivity. Results presented in Sreedevi & Rama Rao (2019) show that RL-LCDC optimizes the connectivity with lower delay and better energy efficiency when compared with other conventional schemes. Although both approaches (Sreedevi & Rama Rao, 2019; Khuntia & Hazra, 2019) produce interesting results, we alternatively propose an algorithm in this paper that is not based on reinforcement learning that can solve the problem with a lower computational complexity.

Song et al. (2019) proposed a joint uplink and downlink (JUAD) resource allocation scheme which maximizes system capacity and guarantees the SINR constraint for CUEs and DUEs. The authors formulate the optimization problem as a mixed integer nonlinear programing problem (MINLP) and divide it into two sub-problems, the power allocation and channel assignment. At first, the optimal transmission power is obtained through the convex objective function. Then, it is developed the Hungarian algorithm to achieve joint uplink and downlink channel assignment, improving system capacity performance and increasing spectrum efficiency. Authors show through simulations that the performance of the algorithm is better than that of schemes for independent allocation. The JUAD algorithm has among its advantages the fact that it works with allocation in the uplink and downlink.

Li et al. (2019) proposed to use a resource allocation scheme that integrates a probabilistic approach to a quasi-convex optimization algorithm based on channel probability statistical characteristics for D2D communication mode selection and resource optimization. The authors also proposed a sub-optimal allocation algorithm when number of users is too large, in order to save costs and improve efficiency. The presented results show that the algorithm optimizes total throughput of the system and reduces communication interference between users. However, in the same way as JUAD and the other mentioned algorithms, the algorithm proposed in Li et al. (2019) does not consider the user’s system delay in the optimization process, an important parameter in any resource allocation system, especially if we consider that D2D communication takes advantage of the proximity between users to increase spectral efficiency. That is, by decreasing communication delays of the links, spectral efficiency can be increased.

## Delay minimization based d2d resource allocation algorithm

In this section, a multi-sharing resource allocation algorithm for D2D communication is proposed which considers the estimated delay information as a utility function to be optimized. To estimate the delay, an approach based on deterministic network calculus concepts is also proposed.

Deterministic network calculus can be used to estimate resources in order to provide quality of service (QoS) in networks and has provided powerful tools for estimating backlog and delay in a network with guaranteed service for individual traffic flows. Using the notion of envelope process, arrival curves and service curves, several studies have shown that the backlog and delay bound can be concisely expressed by the Min-Plus algebra (Le Boudec & Thiran, 2004).

Network calculus can also be seen as the systems theory that applies to computer networks, but the main difference is to consider another algebra (Gonzaga Ferreira & Teles Vieira, 2020).

### MFBAP envelope process

In general, fractals are described in the literature according to a set of behaviors and characteristics, such as self-similarity, phenomena with patterns that are repeated at different scales and with irregular structures. Multifractals are characterized by a set of fractal dimensions and are used to treat phenomena that occur in multiple scales and dimensions (Feldmann, Gilbert & Willinger, 1998).

Network traffic traces captured at small scales in general tend to be multifractal. This means that they present highly dependent structure between samples with burst incidences at various scales. These characteristics can degrade network performance in relation to traffic flows considered Gaussian and short-dependent (Feldmann, Gilbert & Willinger, 1998). In this work, we propose to use a multifractal envelope process once it was shown that it can better describe real traffic envelope processes at the time scale considered in this paper than monofractal based or short-range based envelope processes (Santos & Vieira, 2015).

The envelope process for incoming packet traffic is an upper bound for the actual accumulated packet traffic process. For a deterministic envelope process, the function $\hat{A}(t)$ corresponds to the maximum value of a flow *A*(*t*) in the time slot [*s*, *s* + *t*], and is defined by equation as follows (Le Boudec & Thiran, 2004):

(7) $$\hat{A}(t)=\underset{s\geq 0}{\sup}A\left[s,s+t\right],$$

where sup is an operator that returns the maximum value of *A*[*s*, *s* + *t*] without establishing an upper bound value *s* ≥ 0 in this case.

The multifractal bounded arrival process (MFBAP) is a deterministic alternative to obtain the envelope process that limits the volume of traffic in a given time interval, calculated as follows (Santos & Vieira, 2015):

(8) $${\hat{A}}_{MFBAP}(t)=\bar{a}t+k\sigma t^{H(t)}+B,$$

where *H*(*t*) is the Hölder exponent (Murali Krishna, Gadre & Desai, 2003), that represents the degree of the singularity of the function, *t* is the instant of time, ā and σ are respectively the mean and standard deviation of incoming traffic, *k* is the constant related to the probability of violation (for *ε* = 10^−6^) of the envelope process and *B* is the buffer size.

### Delay bound estimation

The service curve concept has been explored as estimation tool involving various technologies and scenarios, mainly in the area of deterministic and statistical network calculus (Santos & Vieira, 2015). The advantage of the network calculus theory is due to the very intuitive convolution formulas that can be used to determine traffic output processes of a communication system from its arrival envelope process and service curve (Le Boudec & Thiran, 2004).

The upper bound on delay, denoted by $\hat{d}$, is given by Santos & Vieira (2015):

(9) $$\hat{d}=\inf\left\{d\geq 0|\forall t\geq 0:A^{*}(t-d)\leq S(t)\right\},$$

where inf is an operator that returns, in this case, the lowest value of *d* ≥ 0 obeying A* (*t* − *d*) ≤ *S*(*t*). A* is the MFBAP envelope process, calculated according to Eq. (8) and *S* is the generalized service curve of an OFDM (orthogonal frequency-division multiple access) system for any user served for the same time interval *T*, and can be denoted as Costa (2013):

(10) $$S_{n_{S}}(t)=cTP+cT\min\left\{\frac{\max\left[t-PN_{S}T-(n_{S}-1)T;0\right]}{T};1\right\},$$

where *c* is the average service rate on the system server and *N*_*S*_ is the number of time slots *T* per complete cycle *P* given by $P=\left\lfloor \frac{t}{N_{S}T}\right\rfloor$. The operator represents the smallest integer closest to $\frac{t}{N_{S}T}$. The use of service curve allows to obtain network performance and behavior parameters in an analytical way, as well as estimates of the delay and backlog bounds.

### Delay minimization conflict graph algorithm

In this section, we propose a resource allocation algorithm for the uplink of communication system with D2D users that considers the minimization of delay, called DMCG (delay minimization conflict graph) algorithm. It is proposed in this paper to use Eq. (9) to estimate the delay due to its precision as verified in previously works such as Santos & Vieira (2015) and Gonzaga Ferreira & Teles Vieira (2020), which turns it possible to make early decisions on wireless networks resource scheduling.

The DMCG algorithm is started by randomly allocating idle RBs, i.e., one DUE pair is randomly chosen for each idle RB. Then, the algorithm decides how to reuse RBs of all CUEs. A group of unmarked pair *m* is formed by adding each unallocated DUE pair *n* to it in order to optimize utility function (9), forming a set denoted by *γ*_*m*_. A set of DUE pairs that maximize the total utility is contained into the largest group *m*′ with unallocated DUE pairs, taking the maximum weight independent set of the conflict graph corresponding to group *m*′. It is allowed that DUE pairs reuse RBs of CUE *m*′. Candidates are removed one by one until the SINR requirements are met. Then, finishing an iteration, the set of DUE pairs that will reuse RBs of CUE *m*′, denoted as Δ_*m*′_, ends up being the remaining candidates and group *m*′ is marked done. Another iteration is effectuated if there are unmarked groups. Thus, the DMCG algorithm works in an iterative way by forming conflict graphs *G*_*m*′_ and seeking to meet the SINR requirements at each iteration

The set of vertices of the conflict graph *G*_*m*′_ corresponds to the DUE pairs in group *m*′. A weight value is assigned to each vertex according to the utility of the corresponding DUE pair when it joins group *m*′. In the conflict graph, an edge is added for every two vertices if the mutual interference does not meet the threshold. The same RB could not be reused by two DUE pairs. This explains why the edge connects two vertices.

The conflict graph *G*_*m*′_ formed by DUE pairs representing the vertices helps to determine the candidates. Thus, the objective is to remove the DUE pairs that cannot coexist due to the large mutual interference and to keep the DUE pairs that maximize the throughput of the system. Candidates are chosen as the maximum weight independent set of the conflict graph *G*_*m*′_. Finally, the set Δ_1_,Δ_2_,…,Δ_*M*_ of DUE pairs *n* that reuse RBs allocated to CUE *m* is returned as the algorithm result.

Algorithms 1 and 2 show the pseudo-code for the proposed multi-sharing resource allocation algorithm, an efficient solution to the maximization problem described by Eq. (3) and subject to constraints (4), (5) and (6).

**Algorithm 1:** Delay minimization conflict graph algorithm.

**Data:** *M* CUEs, *N* DUE pairs, *K* idle RBs and *M*−*K* real CUEs;
**Result:** Δ_1_,Δ_2_,…,Δ*_M_* (set of the DUE pairs that reuse RBs allocated to CUE m);
1 For each idle RB, randomly pick *K* DUE pairs $D_{\delta_{1}},D_{\delta_{2}},\ldots ,D_{\delta_{K}}$ ;
/* *U* is the set of unmarked groups */
2 *U* ←{1,2,…,*M*};
/* Γ is the set of DUES that joins group *m* */
3 Γ_1_,Γ_2_,…,Γ*_M_* ← 0;
/* Initialize */
4 **for** *n* ∈ {1,2,…,*N*}−{δ_1_,δ_2_,…,δ*_K_*} do
/* Call Algorithm 2 */
5 m* ← *OptimizeDelay*(*n*,*U*);
/* *D_n_* joins group *m*′ */
6 Γ_m*_ ← Γ_m*_ ∪{*n*};
7 **end**
/* Main body */
8 **while** *U* ≠ 0 **do**
9 Form the conflict graph *G_m′_* for the largest group Γ_*m*′_ in *U*;
10 Δ_*m*′_ ← maximum weight independent set of *G_m_*_′_ ;
11 **for** *n*′ ∈ Δ_*m*′_ **do**
/* Check if DUE pair *n*′ meet the SINR requirement */
12 **if** $P_{m\prime }G_{m\prime n\prime }+\Sigma_{n}\epsilon \Delta_{m\prime }-\{n\prime \}P_{n}G_{nn\prime }\geq I_{n\prime }$ **then**
13 Remove *n′* from Δ_*m*′_ ;
14 **end**
15 **end**
16 In descending order, sort DUEs in Δ_*m*′_ by their interference on *C_m_*_′_ ;
/* Remove one DUE from Δ_*m*′_ until the maximum tolerable interference *I_m_*_′_ constraint is met */
17 **while** $\Sigma_{n\epsilon \Delta m\prime }P_{n}G_{nB}\geq I_{m\prime }$ **do**
18 Remove the first element from Δ_*m*′_;
19 **end**
20 **for** *n* ∈ Γ_*m*′_ − Δ_*m*′_ **do**
/* Call Algorithm 2 */
21 *m*′ ← *OptimizeDelay*(*n*,*U* −{*m*′});
/* *D_n_* joins group m* */
22 Γ_m*_ ← Γ_m*_ ∪{*n*};
23 **end**
/* Group *m*′ is marked */
24 *U* ← *U* −{*m*′};
25 **end**

**Algorithm 2:** Proposed algorithm: delay optimization.

**Data:** DUE *n* and set C;
**Result:** Optimum group m*;
/* Initialize */
1 *OptimumDelay* ← 0;
2 m* ← 0;
/* Calculate m* */
3 **for** *m* ∈ *C* **do**
/* Check the constraint */
4 **if** *P_n_G_nB_* ≤ *I_m_* **then**
/* Estimate delay bound */
5 Calculate $\hat{d}(m)$ according to equation (9);
/* Optimize delay */
6 **if** $\hat{d}(m)$ ≤ *OptimumDelay* **then**
7 *OptimumDelay* ← $\hat{d}(m)$;
8 m* ← *m*;
9 **end**
10 **end**
11 **end**

The operation of Algorithm 1 is similar to that of the GTM+ algorithm, but with the difference of using the estimated delay function described by Eq. (9) instead of a utility function based on throughput. The proposed algorithm seeks to find a solution that minimizes the delay bound estimated to each user and simultaneously meeting the constraints imposed by mutual interference.

The proposed multi-sharing allocation method for uplink transmission described in Algorithm 1 can not be directly applied for a downlink transmission scenario. For this, it would be necessary to reformulate the problem described by Eq. (3) and subject to constraints (4), (5) and (6). This issue will be addressed in future works. Notice also that the DMCG algorithm deals with the reuse of idle resource blocks of CUEs by DUEs, that is, its focus is on determining pairs of DUEs in the network. The joint use of the method proposed in this paper with other techniques available in the literature for resource allocation between devices and base station would solve the allocation problem in a generalized allocation scenario and will be investigated later.

## Simulations and results

This section presents the simulation parameters of the wireless network and the channel modeling used in the simulations, as well as the obtained results.

### Channel modeling for mmWaves

Recent studies developed by 3GPP regarding high frequency bands between 0.5 and 100 GHz defined two channel models for this scenario: clustered delay line (CDL) and tapped delay line (TDL). These channel models are applicable for system-level simulations subject to the following conditions (European Telecommunications Standards Institute (ETSI), 2017):

- For system-level simulations, the supported scenarios are microcell and macrocell urban, indoor office and macrocell rural;
- Bandwidth is supported up to 10% of the center frequency, but not greater than 2 GHz.

**Figure 2:**
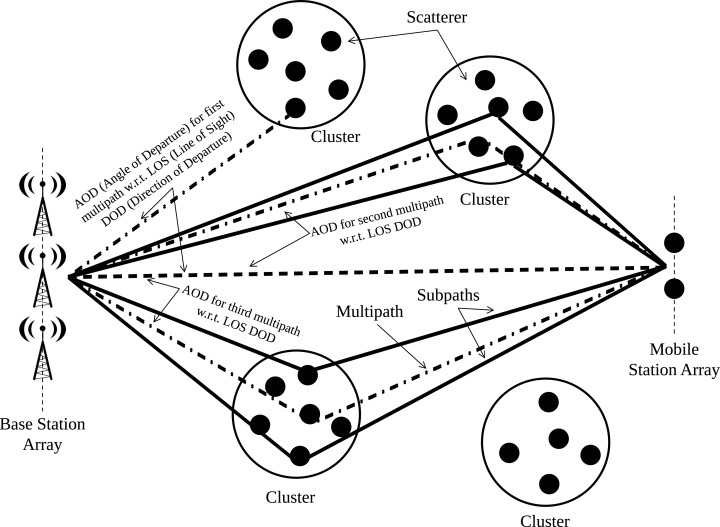
Representation of clusters for CDL model.

The CDL model is a type of channel modeling where the received signal is composed of several separate clusters in delay, as shown in Fig. 2. Each cluster contains a number of multipath components with the same delay value, but with different arrival and departure angles.

CDL models are defined for the frequency range between 0.5 GHz and 100 GHz with a maximum bandwidth of 2 GHz. CDL models can be implemented through the generation of coefficients or the generation of TDL model using spatial filtering.

Three CDL models, called CDL-A, CDL-B and CDL-C, are defined to represent three different channel profiles for non-line of sight (NLOS) environments, while CDL-D and CDL-E models are built for line of sight (LOS) environments (European Telecommunications Standards Institute (ETSI), 2017).

It is considered in this work the multipath model clustered delay line A (CDL-A) (European Telecommunications Standards Institute (ETSI), 2018a, 2018b) , suitable for the mmWaves scenario, and the Rayleigh fading in order to verify the channel modeling impacts via simulations. The carrier frequency of 26 GHz was chosen based on recent studies by Brazilian National Telecommunications Agency (ANATEL) with this frequency range, which should also be adopted in Europe (Tecnoblog, 2019).

### System parameters and results

The simulations were conducted using MATLAB software version R2018a and a microcomputer with the following configuration: Intel Core i7-4785T CPU 2.20 GHz, 8 GB RAM, SSHD SATA III and Windows 10 64 bits. The simulation functions and routines were implemented instead of using available network simulation tools in order to have more control on the simulation scenario configuration and CDL channel modeling parameters than some commercial softwares.

It was compared the simulation results of the proposed DMCG algorithm with those of the greedy throughput maximization plus (GTM+) presented in Ciou et al. (2015), a genetic algorithm (GA) based approach, which aims to find a solution for the maximization problem described by Eq. (3) subject to constraints (4), (5) and (6), and a random reuse scheme. The GA-based algorithm was developed using 30 individuals and 100 iterations.

All CUEs and DUE pairs are randomly distributed in a single cell with the BS at the center and are set to have the same parameters such as SINR threshold, transmission power and noise spectral density, varying only the location in the cell. Each figure reports simulation results averaged over 1,000 transmission time intervals (TTIs) in order to reflect the average system performance. The system simulation parameters are given in Table 2. Most parameter values were set according to references (Sun et al., 2013) and (Ciou et al., 2015).

**Table 2:** Simulation parameters.

Multipath models	Rayleigh and CDL-A
CUE transmission power	23 dBm
DUE transmission power	10 dBm
SINR requirement of each CUE	7 dB
SINR requirement of each DUE pair	4.7 dB
Radius of BS coverage	500 m
Distance between each DUE pair	15 m
White noise power density	−174 dBm/Hz
Path loss model for CUE and DUE	128.1 + 37.6 *log*10 (*d* [*km*])
Path loss model for DUE pairs	148 + 40 *log*10 (*d* [*km*])
System bandwidth	20 MHz
Number of RBs	100
Carrier frequency	26 GHz
Number of real CUEs	40
Number of CUEs	50 until 100
Number of DUE pairs	160
Number of TTIs	1,000

In this work, it is simulated an uplink based on CP-OFDM (cyclic prefix - orthogonal frequency-division multiple access) whose configuration consists of subcarrier spacing of 15 kHz and normal cyclic prefix (CP), as described in European Telecommunications Standards Institute (ETSI) (2018b). Each radio frame occupies 10 ms, divided in 10 subframes of 1 ms, and each subframe is divided in two time slots of 0.5 ms with 7 symbols for each time slot when using normal CP. In the frequency domain the resources are aggregated in 12 subcarriers with 15 kHz bandwidth, totalizing 180 kHz bandwidth, defined as a resource block (RB), the basic unity of resource allocation.

Five traces of real transmission control protocol/Internet protocol (TCP/IP) network traffic were used to represent users’ data traffic during the simulation of the algorithms, which were aggregated in the time domain at 1 ms intervals and assigned to users randomly. These series represent TCP/IP traffic between the University of Waikato (2020) and external networks and were collected between 20/05/2011 and 29/10/2011.

**Figure 3:**
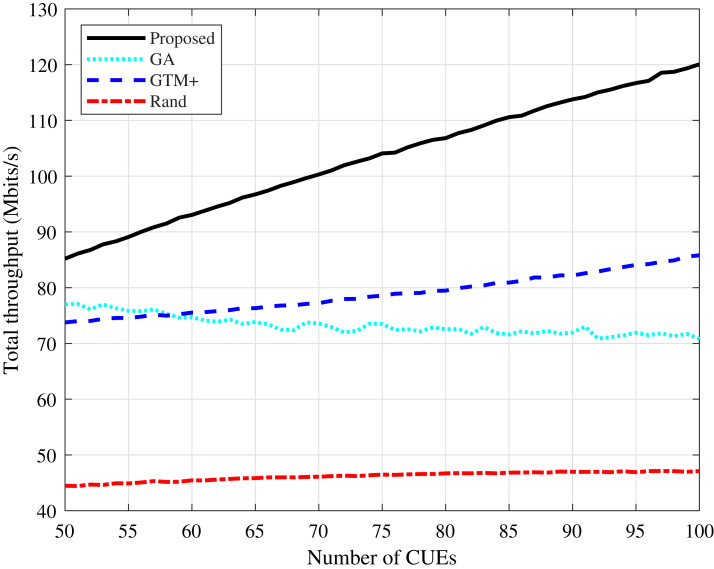
CDL-A channel model: total throughput for different number of CUEs.

**Figure 4:**
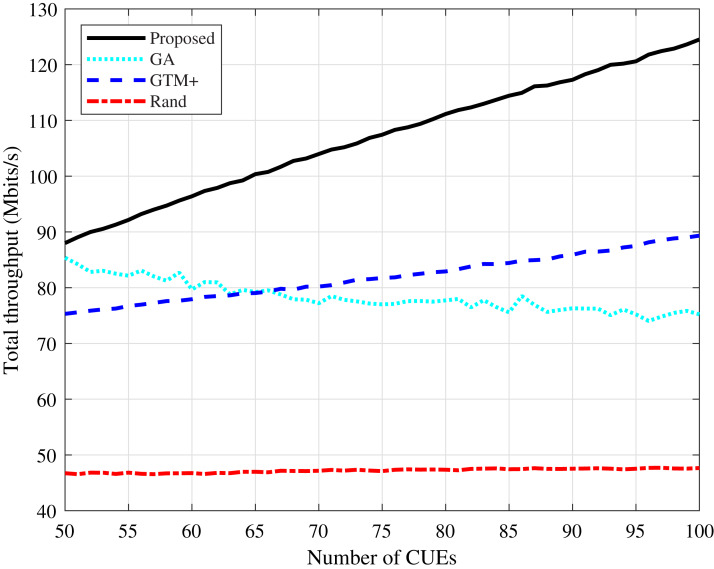
Rayleigh channel model: total throughput for different number of CUEs.

The total throughput values of the system calculated according to Eq. (3) and the sum of all devices throughput are shown in Figs. 3 and 4. It can be seen that the proposed algorithm has the highest values in the two simulated scenarios, both with Rayleigh and CDL-A modeling. The GTM+ and the DMCG algorithms presented higher throughput values compared to the GA-based allocation, showing that the conflict graph strategy is more efficient than solving the problem represented by Eqs. (3)–(6) by directly applying optimization algorithms. The proposed DMCG outperforms the GTM+ because it is considered the conflict graph strategy in conjunction to delay minimization. In this way, in order to reduce system delay, the proposed DMCG algorithm must increase system throughput similar as done by the GTM+ or find a solution that optimizes the allocation process yielding the system delay minimization. The performance of the proposed algorithm improves as the number of CUEs in the network increases, while the performance of the GA-based algorithm tends to become worse.

**Figure 5:**
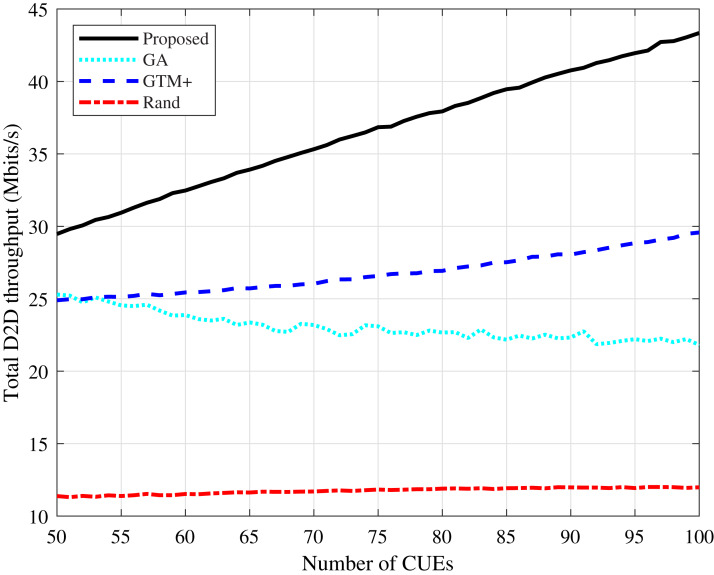
CDL-A channel model: total D2D throughput for different number of CUEs.

**Figure 6:**
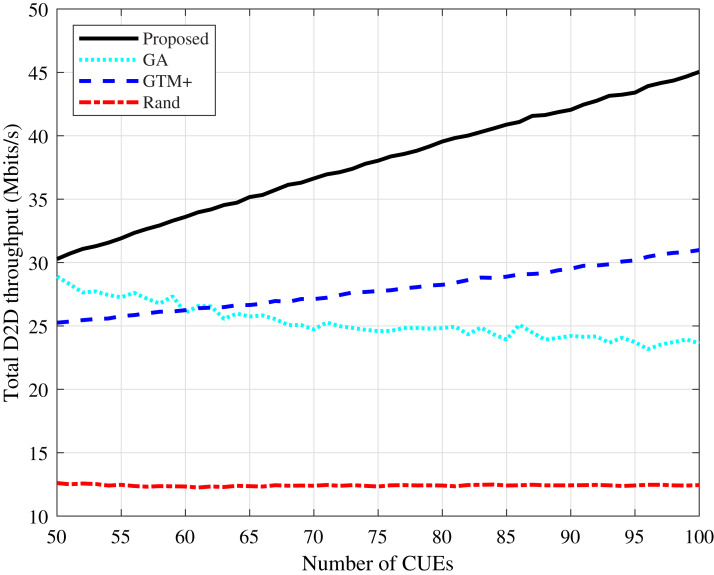
Rayleigh channel model: total D2D throughput for different number of CUEs.

It can be observed a similar behavior regarding the D2D devices throughput, as shown in Figs. 5 and 6. The proposed algorithm improves considerably in terms of throughput compared to the GTM+ algorithm.

**Figure 7:**
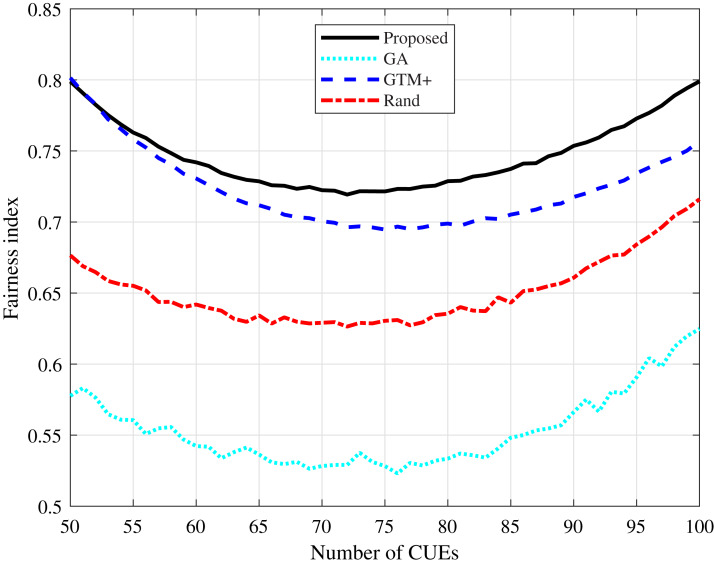
CDL-A channel model: fairness index for different number of CUEs.

**Figure 8:**
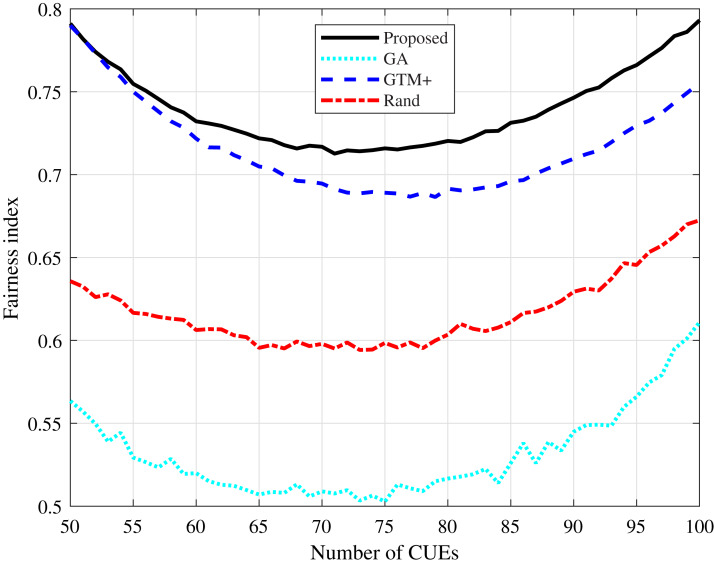
Rayleigh channel model: fairness index for different number of CUEs.

Fairness is an important measure that determines how fair the distribution of resources among system users is, calculated as described in Jain, Chiu & Hawe (1998). It can be seen in Figs. 7 and 8 that the proposed algorithm presents the highest fairness values in all simulated scenarios, showing that it presents a fair distribution of resources among the users of the system. The algorithm based on GA presents the lowest fairness values, a fact expected once the algorithm considers only the system total throughput and the interference constraints, not taking into account a fair distribution of resources.

**Figure 9:**
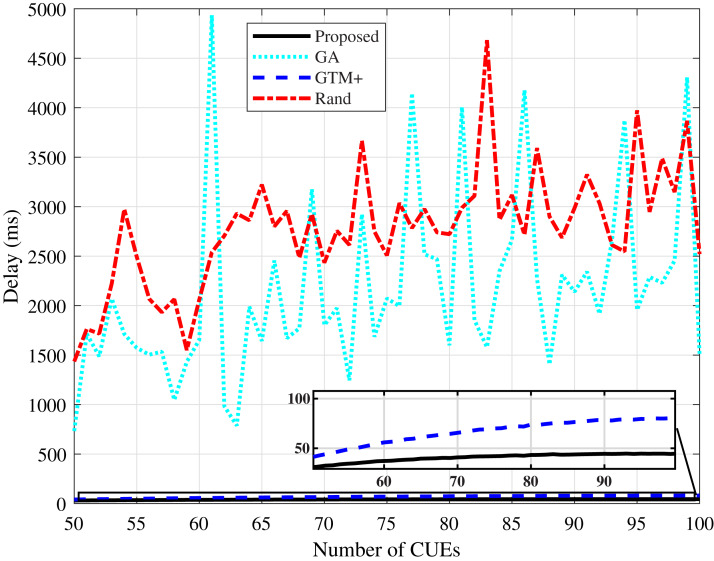
CDL-A channel model: mean delay for different number of CUEs.

**Figure 10:**
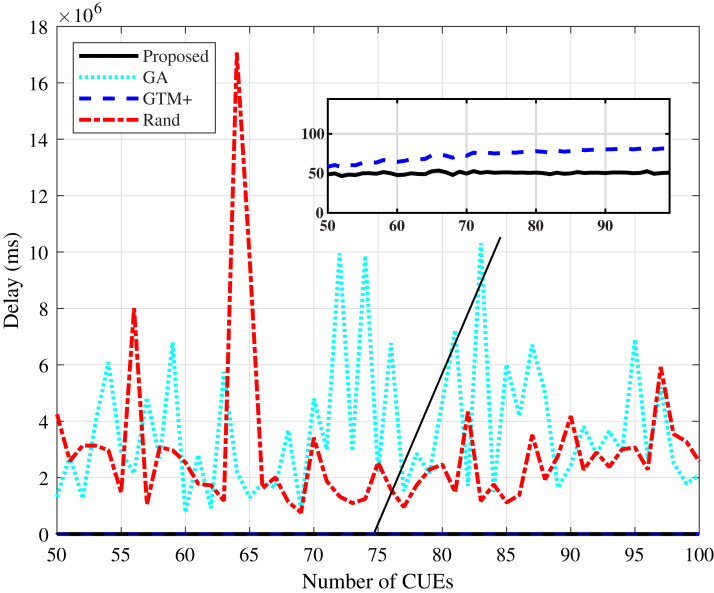
Rayleigh channel model: mean delay for different number of CUEs.

Figures 9 and 10 present the average delay values calculated for the compared algorithms. The values presented by the proposed DMCG algorithm are the lowest values, even lower than the values presented by the GTM+ algorithm. This result proves that the DMCG algorithm is efficient in its strategy of using the estimated delay as a utility function in the allocation system, resulting in a considerable drop in the delay value in both simulated scenarios. The delay values of the GA-based algorithm and the random algorithm vary widely in the simulated scenarios. In the tests, the presence of these peaks of values was verified due to the characteristic of these algorithms of not distributing resources in a fair way, i.e., the characteristic of penalizing some users in terms of delay in detriment of total throughput of the system.

The performance improvement of the proposed algorithm in relation to the other studied algorithms is mainly due to its characteristic of reducing the average delay of the system through the verification of the utility function defined by the estimated delay parameter. The reduction of the average delay of the system has a direct consequence in the improvement of the system throughput and in the fairness parameter.

**Figure 11:**
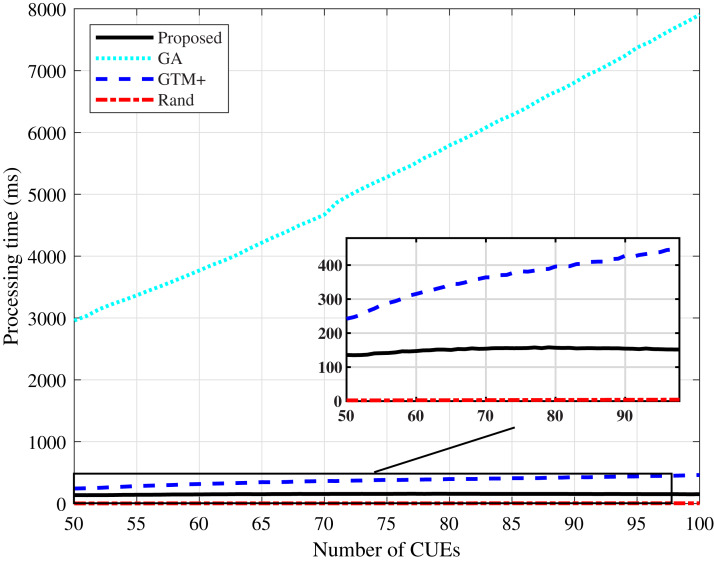
CDL-A channel model: processing time for different number of CUEs.

**Figure 12:**
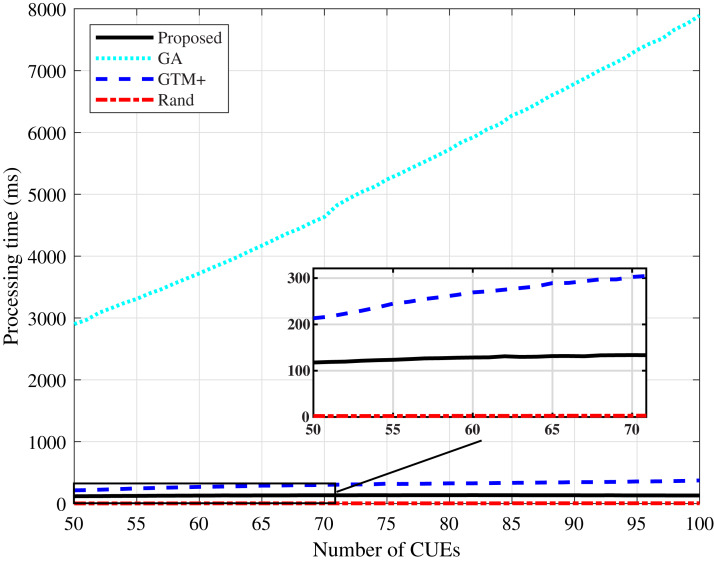
Rayleigh channel model: processing time for different number of CUEs.

Regarding the processing time, shown in Figs. 11 and 12, it can be noticed that the algorithm based on GA has the highest values. GA-based heuristics is the one that demands the most processing among the compared algorithms, due to the large number of variables involved in the system. The processing time tends to increase as the number of CUEs in the network increases. The proposed DMCG algorithm had a considerable performance improvement in processing time when compared to the GTM+ algorithm, although both have computational complexity *O*(*n*^4^). The simulation results confirm that the proposed algorithm based on delay minimization outperforms the GTM+ algorithm for all considered performance parameters.

## Conclusion

This paper presented a multi-sharing resource allocation algorithm for device-to-device (D2D) communication networks in a scenario with millimeter waves above 6 GHz. The proposed DMCG algorithm considers the minimization of a delay function estimated using concepts of network calculus such as traffic envelope process and service curve to decide on the allocation of idle resources in the network to D2D devices.

The results presented for two channel modeling scenarios (Rayleigh and CDL-A) show that the DMCG algorithm provides the highest throughput values of the system when compared to the algorithms based on GA, GTM+ algorithm and the random algorithm. The proposed algorithm also yields higher values of fairness and lower values of delay, as well as processing time shorter than the processing time presented by the GTM+ and GA-based algorithms.

These results show that the use of estimated delay information proved to enhance the multi-sharing resource allocation performance that is based on conflict graphs and maximal weight independent set, with improvement in all requirements in relation to the GTM+ algorithm and with the advantage of being able to anticipate the allocation of resources in a D2D communication scenario with mmWaves.

As a proposal for future work, we intend to extend the communication scenario considering optimized resource allocation for CUEs devices and the application of a variation of the proposed approach involving downlink data transmission. The use of the proposed algorithm in the downlink transmission could considerably improve spectral efficiency by reusing idle resources of network users.

## Supplemental Information

10.7717/peerj-cs.462/supp-1 Supplemental Information 1 Traces of real TCP IP network traffic. These series represent TCP/IP traffic between the University of Waikato and external networks and were collected between 20/05/2011 and 29/10/2011. They were used to represent users’ data traffic during the simulation of the algorithms.

10.7717/peerj-cs.462/supp-2 Supplemental Information 2 DMCG Algorithm MATLAB code. Algorithms simulated at work and all the necessary functions to run the code.

## Additional Information and Declarations

### Data Availability

The following information was supplied regarding data availability: Raw data and code are available in the Supplemental Files.
